# Benzodiazepine prescribing patterns and drug overdose mortality among individuals receiving opioid analgesics

**DOI:** 10.1186/1940-0640-10-S1-A48

**Published:** 2015-02-20

**Authors:** Tae Woo Park, Richard Saitz, Dara Ganoczy, Mark A Ilgen, Amy SB Bohnert

**Affiliations:** 1 Division of General Internal Medicine, Department of Medicine, Alpert Medical School and Rhode Island Hospital, Providence, RI, 02903, USA; 2 Department of Psychiatry and Human Behavior; Alpert Medical School and Rhode Island Hospital, Providence, RI, 02903, USA; 3 Clinical Addiction Research and Education (CARE) Unit, Section of General Internal Medicine, Department of Medicine, Boston Medical Center and Boston University School of Medicine, Boston, MA, 02118, USA; 4 Department of Community Health Sciences, Boston University School of Public Health, Boston, MA, 02118, USA; 5 Department of Veterans Affairs, Health Services Research & Development (HSR&D), Ann Arbor, MI, 48109, USA; 6 Center of Excellence, and Serious Mental Illness Treatment Resource and Evaluation Center, Ann Arbor, MI, 48109, USA

## Background

Drug overdose mortality, particularly involving opioid analgesics, has risen steadily over the past two decades and is now the leading cause of injury death in the United States. Benzodiazepines are commonly prescribed concurrently to individuals receiving opioid analgesics. Little is known about the association between benzodiazepine-prescribing patterns and overdose mortality in those receiving opioid analgesics. We aimed to study the association between benzodiazepine prescription history, dose, type and dosing schedule, and the risk of drug overdose mortality among individuals receiving opioid analgesics.

## Materials and methods

This was a case-cohort study utilizing Veterans Health Administration (VHA) administrative data. Participants were all individuals who died of a drug overdose (n = 2326) while receiving opioid analgesics and a random sample of individuals (n = 404725) who received VHA medical services and opioid analgesics between fiscal years 2004 and 2009. Benzodiazepine prescription history, dose, type, and dosing schedule were determined by dispensation data from the VHA’s Pharmacy Benefits Management Services. Benzodiazepine prescription history was categorized as periods of time when individuals were currently prescribed, formerly prescribed, or not prescribed benzodiazepines. Drug overdose mortality was based on cause of death information from the National Death Index. Associations between benzodiazepine prescription history, dose, type and dosing schedule, and overdose mortality were examined using Cox proportional hazards models.

## Results

Twenty-seven percent of individuals who received opioid analgesics also received benzodiazepines during the study period. Approximately half of the drug overdose deaths (n = 1162) occurred during periods when individuals were concurrently receiving benzodiazepines and opioids. Risk of overdose death increased based on benzodiazepine prescription history: formerly prescribed versus not prescribed, adjusted hazard ratio (HR) = 2.22 (95% confidence interval [CI]; 1.95–2.52; absolute risk difference approximation [ARDA] = 0.12%); currently prescribed versus not prescribed, adjusted HR = 3.59 (95% CI, 3.24–3.97; ARDA = 0.25%). Risk of overdose death increased as daily benzodiazepine dose increased. When compared to clonazepam, temazepam was associated with a decreased overdose risk; adjusted HR = 0.73 (95% CI; 0.56–0.95; ARDA = 0.03%). Benzodiazepine dosing schedule was not associated with overdose risk.

## Conclusions

Among individuals receiving opioid analgesics, receipt of benzodiazepines was associated with an increased risk of drug overdose death, and these risks were generally consistent at all levels of opioid dose. The risks and benefits of benzodiazepine-prescribing in this population warrant further evaluation.

